# Selective APRIL Blockade Delays Systemic Lupus Erythematosus in Mouse

**DOI:** 10.1371/journal.pone.0031837

**Published:** 2012-02-15

**Authors:** Bertrand Huard, Ngoc Lan Tran, Mahdia Benkhoucha, Céline Manzin-Lorenzi, Marie-Laure Santiago-Raber

**Affiliations:** 1 Department of Pathology and Immunology, University of Geneva, Geneva, Switzerland; 2 Division of Hematology, University Hospital of Geneva, Geneva, Switzerland; Beth Israel Deaconess Medical Center, United States of America

## Abstract

SLE pathogenesis is complex, but it is now widely accepted that autoantibodies play a key role in the process by forming excessive immune complexes; their deposits within tissues leading to inflammation and functional damages. A proliferation inducing ligand (APRIL) is a member of the tumor necrosis factor (TNF) superfamily mediating antibody-producing plasma cell (PC)-survival that may be involved in the duration of pathogenic autoantibodies in lupus. We found significant increases of APRIL at the mRNA and protein levels in bone marrow but not spleen cells from NZB/W lupus mice, as compared to control mice. Selective antibody-mediated APRIL blockade delays disease development in this model by preventing proteinuria, kidney lesions, and mortality. Notably, this was achieved by decreasing anti-DNA and anti-chromatin autoantibody levels, without any perturbation of B- and T- cell homeostasis. Thus, anti-APRIL treatment may constitute an alternative therapy in SLE highly specific to PCs compared to other B-cell targeting therapies tested in this disease, and likely to be associated with less adverse effects than any anti-inflammatory and immunosuppressant agents previously used.

## Introduction

Systemic Lupus Erythematosus (SLE) is a disorder of systemic autoimmunity characterized by the production of autoantibodies and subsequent development of glomerulonephritis (GN). The availability of several mouse strains, which spontaneously develop an autoimmune syndrome resembling human SLE, offers the opportunity to evaluate therapeutic approaches. In human, SLE is usually treated with steroids in combination with cytotoxic compounds that targets cycling cells, such as cyclophosphamide. Although this treatment has been successful in managing SLE, not all patients respond to cyclophosphamide, implying to explore alternative treatments [1]. Important efforts are currently being made to target B cells in this disease (reviewed in [1], [2]). Those treatments include the antagonism of two members of the tumor necrosis factor (TNF) superfamily, the B-cell activation factor (BAFF) and a proliferation-inducing ligand (APRIL). BAFF and APRIL share the transmembrane activator, calcium modulator and cyclophilin ligand interactor (TACI) and the B-cell maturation antigen (BCMA) as common receptors from the TNF-R superfamily [3]. Despite these similarities, BAFF and APRIL are not redundant molecules. At the receptor level, BAFF binds to another unique receptor from the TNF-R family, BAFF-R [4], while APRIL uses heparan sulfate proteoglycans (HSPGs) as co-receptors [5], [6]. Differences were also observed functionally in genetically deficient animals. BAFF deletion resulted in a profound decrease in the mature B-cell compartment [7], while APRIL deletion resulted in a more restricted immune deficiency [8] that could be attributed to plasma cells (PC) [9].

Two different treatments were first tested pre-clinically to antagonize BAFF and APRIL. One consists in the use of a soluble form of TACI, which antagonizes both APRIL and BAFF. The other one is characterized by a soluble form of BAFF-R, which antagonizes only BAFF. Both showed promising results in mouse SLE models with reduction of autoantibody production, decreased proteinuria and better survival [10], [11], [12], [13], [14]. Based on these results, clinical trials were instigated with soluble TACI and a monoclonal antibody against human BAFF, in place of soluble BAFF-R, in SLE patients. Similarly to preclinical experimentations, these treatments showed interesting results (reviewed in [1], [2]). A BAFF blockade clinically as efficient as BAFF/APRIL blockade indicates that APRIL antagonism might be dispensable in SLE. However, APRIL antagonism alone has never been tested so far, because of the lack of a specific reagent. Here, we report the generation of the first mAb blocking mouse APRIL and its effect on lupus spontaneously occurring in NZB/W mice.

## Results

### Increased level of APRIL production in lupus-prone mice

**Figure 1:**
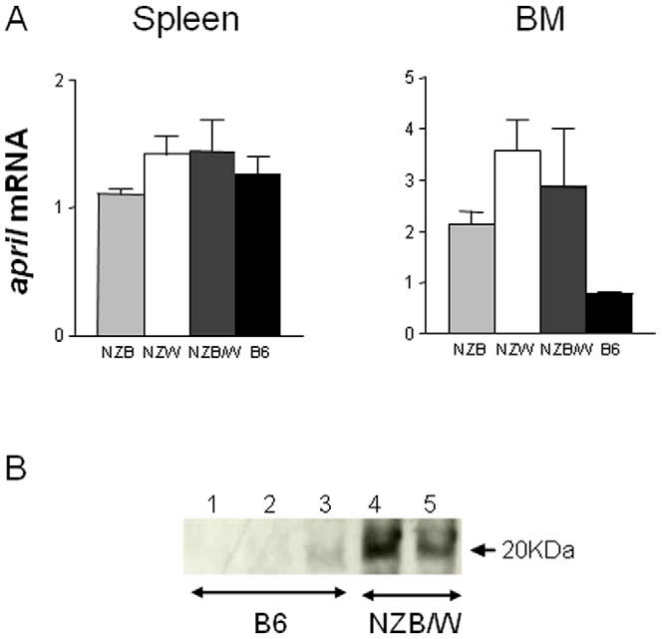
Increased level of APRIL production in BM from lupus-prone mice. (**A**) Levels of *APRIL* mRNA (means ± SD, N = 3) are shown in total spleen and BM cells from the indicated mice. (**B**) Western blot analysis of APRIL in BM supernatants from the indicated mice.

APRIL polymorphisms have been found to play a role in the susceptibility to SLE in the Japanese population [15]. In addition, elevated levels of APRIL in sera or cerebrospinal fluid have been reported in patients with SLE, which correlated with disease activity [16], [17], [18]. In animals such correlation has not been well investigated so far. For that purpose, we assessed whether lupus mice expressed elevated levels of APRIL. The abundance of *april* mRNA in splenic and bone marrow (BM) cells from 5 mo-old NZB/W females, age-matched parental strains NZW males and NZB females and non-autoimmune female B6 control mice was quantified by real-time PCR. We found a 2.5- 4.6- and 3.7-fold increase of *april* mRNA in BM but not spleen cells (*p*≤0.01) from NZB, NZW and NZB/W mice, respectively, as compared with those from B6 females (Figure 1A). We further confirmed APRIL upregulation by showing an increase of soluble APRIL in supernatants obtained from BM flushes of NZB/W mice (Figure 1B).

### Characterization of the anti-APRIL blocking antibody

**Figure 2:**
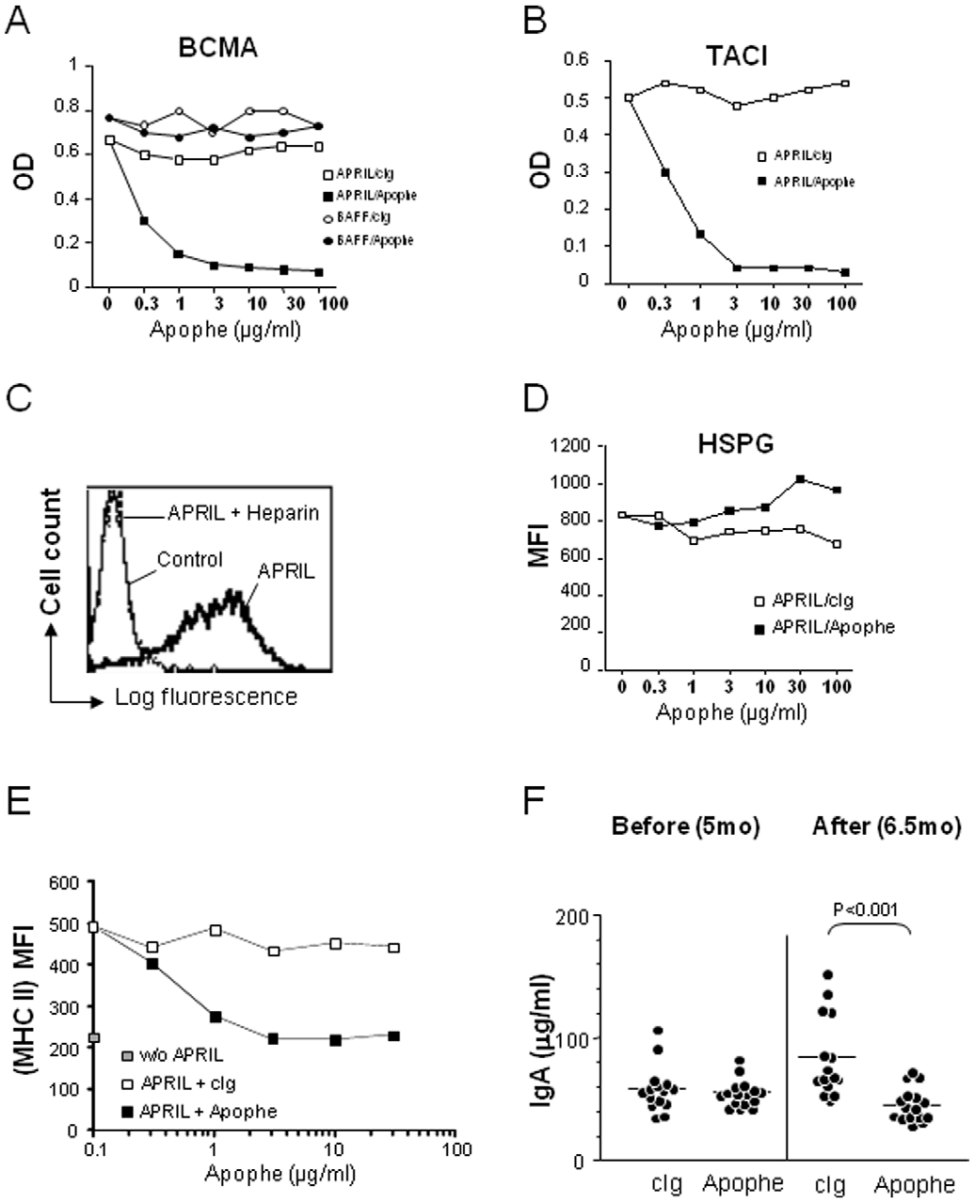
Blocking anti-APRIL antibody features. (**A**) The interaction of APRIL or BAFF with plastic-coated BCMA-Fc was measured in the presence of the indicated inhibitors by ELISA. (**B**) The interaction of APRIL with plastic-coated TACI-Fc was measured in the presence of the indicated inhibitors. CIg (3a1) was irrelevant isotype-matched control for Apophe. (**C, D**) The binding of APRIL on L363 was measured by flow cytometry, in the presence of the indicated inhibitors. Ctrl (ACRP30) was irrelevant control for APRIL. Data are representative of three experiments. (**E**) Total splenocytes were incubated in medium alone or in the presence of APRIL and the indicated mAbs for 2 days. Mean fluorescence intensity is shown on gated B220^+^ B cells. (**F**) Serum total IgA levels were evaluated. Each symbol represents an individual animal (six-weeks isotype control- or Apophe-treated NZB/W 6.5 mo-old female mice). Results are expressed as µg/ml. The mean values are indicated by the horizontal line.

We selected the anti-mouse APRIL IgG1 mAb, Apophe, for its efficiency (µg/ml range) to block interactions of soluble APRIL with BCMA and TACI (Figure 2A, B). This inhibition was specific to APRIL, since Apophe did not modulate the binding of soluble BAFF to coated BCMA. APRIL binding to a L363 myeloma cell line was highly dependent on APRIL/HSPG interactions, since it was completely inhibited by the low molecular weight HSPG, heparin (Figure 2C). In this assay, Apophe did not inhibit APRIL/HSPG interactions (Figure 2D). As an *in vitro* cellular assay, we monitored APRIL-mediated MHC class II upregulation on B cells. Figure 2E shows a blockade of MHC class II upregulation in the presence of Apophe. To test the *in vivo* activity of Apophe, we treated female NZB/W mice with 100 µg i.v. of Apophe or control mouse IgG (cIg) twice a week during 6 weeks starting at 5 months of age, and studied serum levels of IgA, the antibody isotype the most affected in APRIL-deficient mice [9]. As shown in figure 2F, anti-APRIL treated mice showed reduced IgA levels compared to controls. Taken together, these results show that Apophe antagonizes mouse APRIL both *in vitro* and *in vivo*.

### APRIL blockade leads to diminished disease manifestation in NZB/W mice

**Figure 3:**
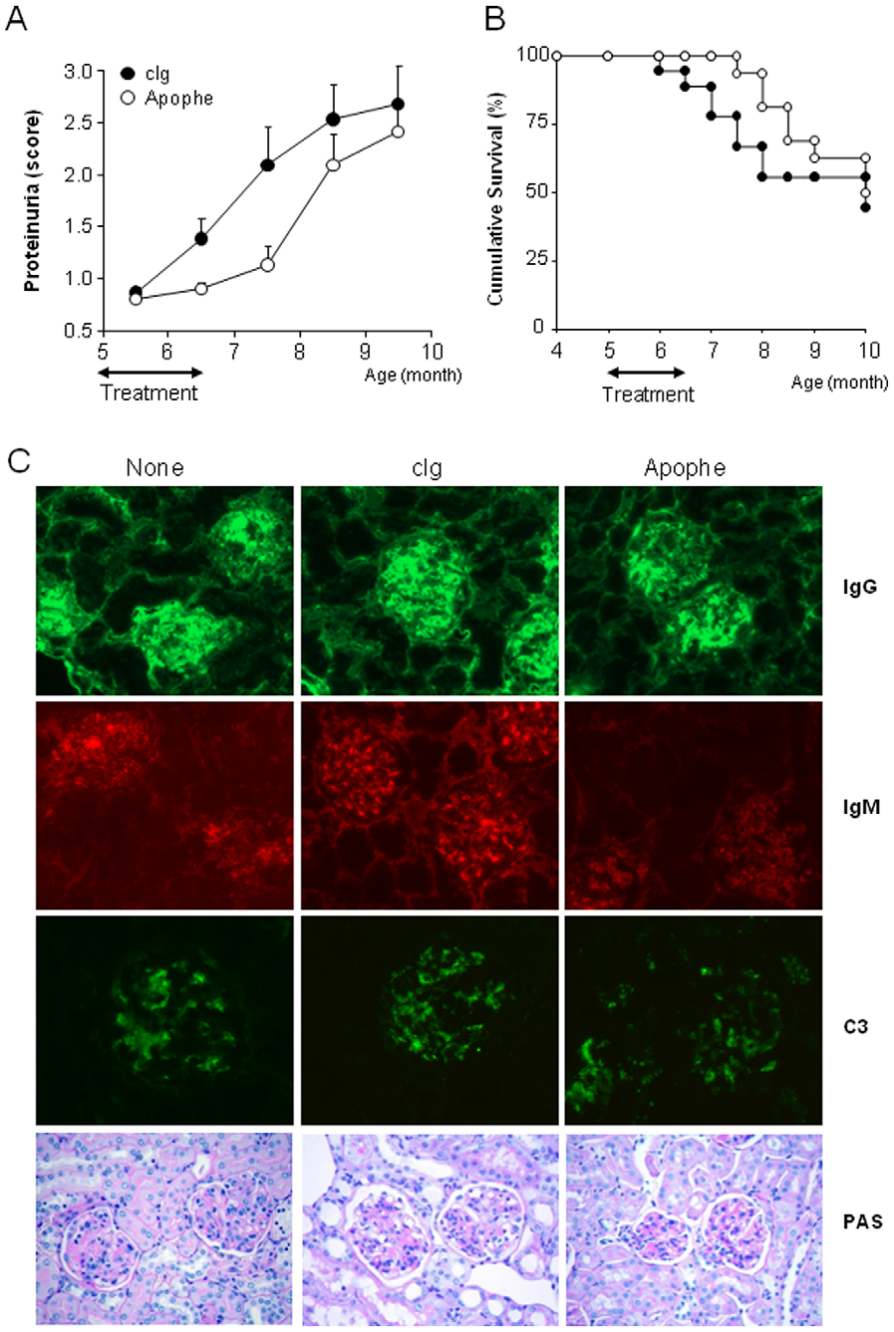
A blocking anti-APRIL antibody delays disease occurrence in NZB/W mice. (**A**) The intensity of proteinuria was scored on a 0 to 4 scale. Mean results are shown. (**B**) 18 isotype control-treated NZB/W (filled circle) and 16 Apophe-treated NZB/W (open circle) female mice were followed for GN-induced mortality. (**C**) Representative histological appearance of kidney glomerular lesions from untreated NZB/W, isotype-control treated and Apophe-treated NZB/W female mice (representative of 3 mice/group). Kidneys sections were stained with either fluorescent anti-IgG, anti-IgM or anti-C3 mAbs or by PAS for histopathology. Note severe glomerular lesions in untreated and isotype-treated mice, which contrast with only limited glomerular alterations (focal increases in mesangial cellularity) in Apophe-treated NZB/W mice at 8 months of age (PAS; original magnification, ×400).

We next determined the effect of APRIL blocking on the development of SLE, in the same groups of NZB/W mice. The treatment was started at 5 months of age, i.e. before any clinical sign of kidney diseases, but after the appearance of autoantibodies in sera. Apophe-treated mice displayed a significant reduction of proteinuria during the treatment at 6 months of age that persisted 6 weeks after treatment stop, at 7.5 months of age, compared to isotype-treated mice (means ± SEM, 6 mo: 0.9±0.1 vs. 1.4±0.2, *p*<0.05; 7 mo: 1.1±0.2 vs. 2.1±0.4, *p*<0.05) (Figure 3A). Consistent with the delayed development of proteinuria, only 6% (1/16) of Apophe-treated mice died at 8 months of age, which contrasted to the higher mortality rate (33%, 6/18) in isotype-treated mice (p<0.05) (Figure 3B). Apophe-treated mice developed disease and reached the same mortality rate than isotype-treated control mice 8 weeks after treatment stop. By 8 months of age, 3 mice per group were sacrificed, and kidneys were stained for IgM, IgG and C3 deposits. As depicted in Figure 3C, we observed less IgM deposits in kidneys from Apophe-treated mice. No clear difference was observed for IgG and C3. Beneficial effect of Apophe treatment, compared to untreated or isotype-treated mice, was also characterized by decreased glomerular cellularity, less deposits of PAS-positive materials, and no glomerular increased size.

### *In vivo* APRIL-targeting reduces IgM and IgG autoantibody production without modulating the mature T- and B-cell repertoire

**Figure 4:**
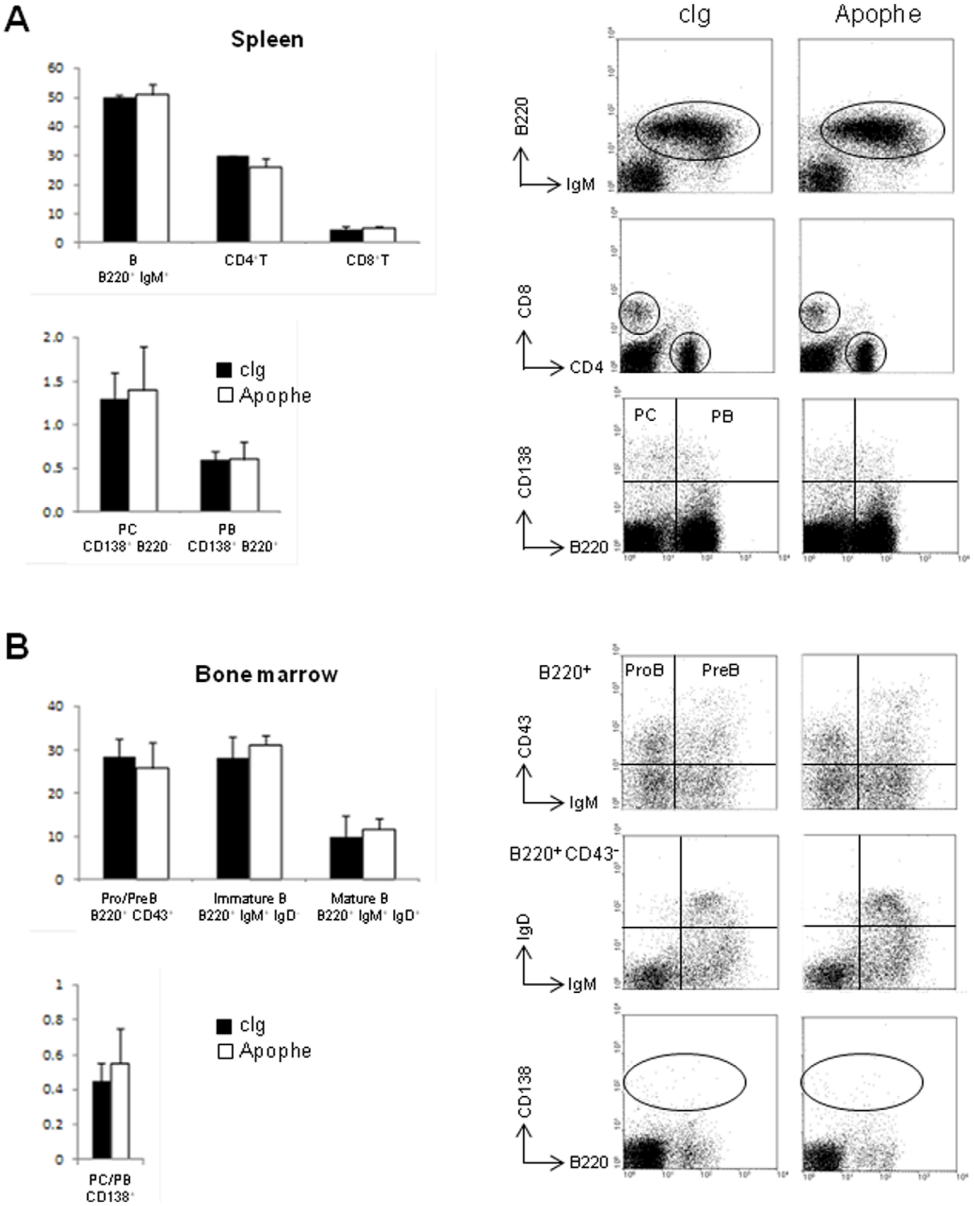
A blocking anti-APRIL does not modulate the T- and B-cell repertoire of NZB/W mice. (**A, B**) Frequencies of the indicated cell populations in spleen (**A**) and BM (**B**) from treated-mice (N = 3 per group) analyzed by flow cytometry at 6 weeks after treatment. Representative flow cytometry profile for each population is shown. PC, plasma cell and PB, plasmablast. Pro/PreB are expressed as frequency of total B220^+^ B cells, immature and mature B cells as frequency of B220^+^ CD43^−^ non-precursors B cells.

We next assessed the immune status of Apophe versus control-treated mice. Total numbers of spleen and bone marrow cells were not different in both groups (means ± SEM, spleen: 246±11×10^6^ in Apophe-treated mice vs. 200±21×10^6^ in isotype-treated mice, *p*>0.05; BM: 22.9±7.4×10^6^ in Apophe-treated mice vs. 20.8±1.7×10^6^ in isotype-treated mice, *p*>0.05 (N = 4 mice per group)). Analysis of splenic cell populations indicated comparable frequencies of total B cells, CD4^+^ and CD8^+^ T cells in control and Apophe-treated mice (Figure 4A). In addition, we observed no difference in percentages of B cell precursors in BM from isotype- and Apophe-treated mice (Figure 4B). We also did not find any differences in the frequencies of plasmablasts/PC in spleen and BM. This is consistent with the normal Ig levels, with the exception of IgA, reported in serum from *APRIL^−/−^* mice [9], and the unaltered numbers of plasmablasts/PC we observed in spleen and BM from (4 month-old) B6.*APRIL^−/−^* mice in the steady state situation (means ± SEM, spleen: 0.63±0.07% in *APRIL^−/−^* vs. 0.64±0.02% in WT mice, *p*>0.05; BM: 0.16±0.02% in *APRIL^−/−^*vs. 0.28±0.06% in WT mice, *p*>0.05 (N = 4 mice per group)).

**Figure 5:**
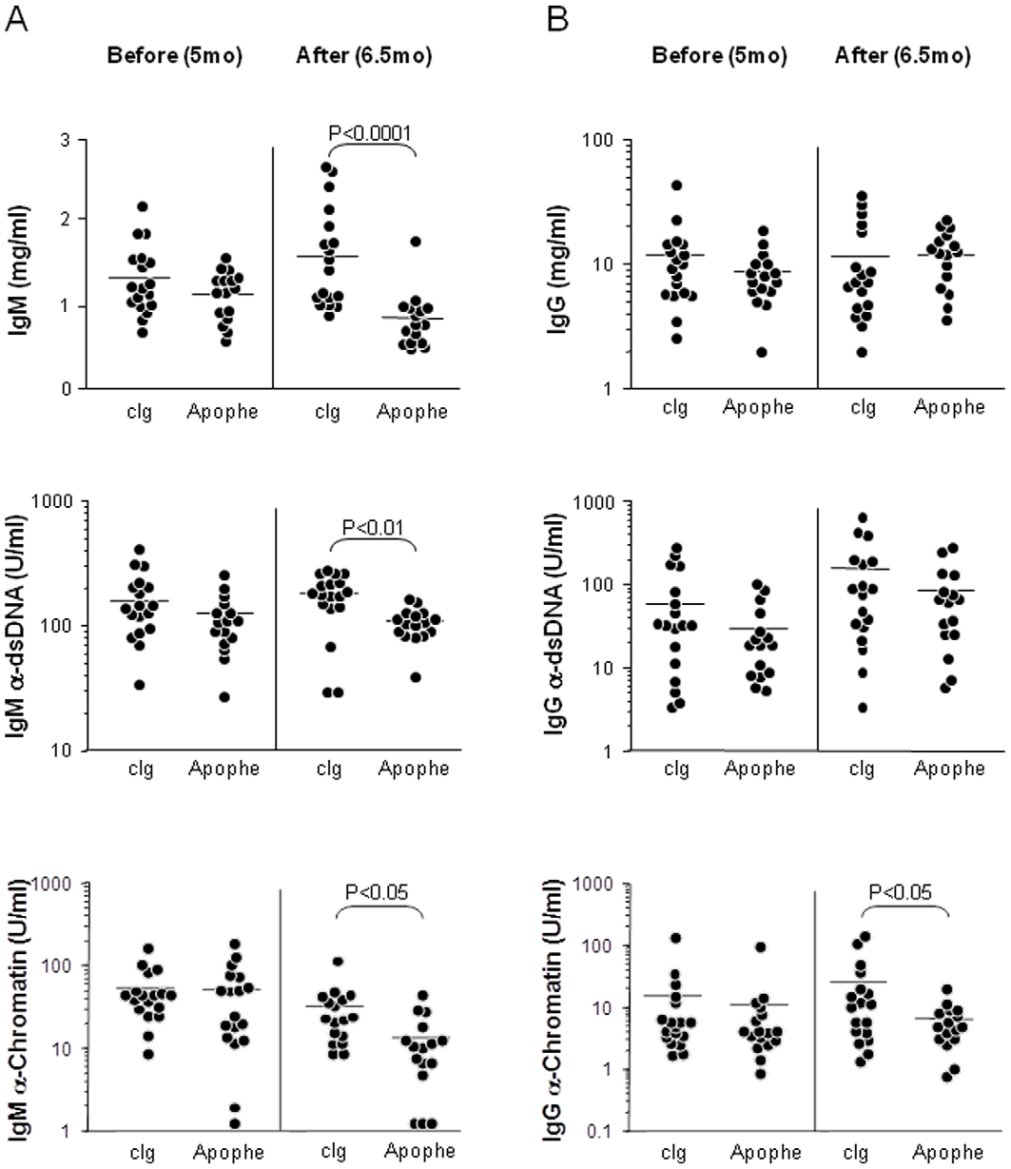
A blocking anti-APRIL antibody decreases autoantibody serum levels. (**A, B**) Each symbol represents an individual animal (18 isotype-treated control NZB/W (cIg) and 16 Apophe-treated NZB/W female mice, before (5 mo.) and after treatment (6.5 mo.). Results are expressed as mg/ml for total IgM and IgG and as U/ml for anti-dsDNA and anti-chromatin autoantibodies. The mean values are indicated by the horizontal bar.

As for IgA, the total IgM levels were reduced at the end of Apophe treatment compared to pre-treated levels (Figure 5A, upper panel). Notably, a decrease in IgM autoantibodies against native DNA (dsDNA) and chromatin compared to cIg-treated mice (Figure 5A, middle and bottom panels) was observed. In contrary no effect in the total IgG serum levels was observed during the 6 weeks period (Figure 5B upper panel). Although serum levels of IgG anti-dsDNA were not affected (Figure 5B middle panel), we observed substantial decrease in serum concentrations of IgG anti-chromatin (means ± SEM, 7.3±1.4 vs. 26.3±9.8 U/ml, *p*<0.05; Figure 5B bottom panel) in treated animals. We thus show that *in vivo* APRIL-targeting leads to reduction of total IgM, IgM and IgG autoantibodies. The effect on IgG autoantibodies was dependant on the specificities.

## Discussion

APRIL upregulation has been observed in SLE patients. Here, we show that the increased APRIL level observed in lupus mice was detectable in the BM, and not in a peripheral lymphoid organ such as the spleen. Several types of cells, mostly belonging to the myeloid lineage, produce APRIL. In secondary lymphoid organs, neutrophils, monocytes and dendritic cells are the dominant cells producing APRIL [19], [20], while myeloid precursor cells constitute an important source of APRIL in BM [21], [22], [23]. Hence, increased myelopoiesis in BM may be part of the immune dysregulation occurring in lupus. Such increased myelopoiesis has been directly reported in lupus mice [24] or indirectly, by elevated monocytosis occurring in blood from lupus animals [25], [26], [27].

With our mAb targeting approach, we are showing for the first time that APRIL has a role by itself on lupus pathology. Indeed, APRIL selective blockade for six weeks after disease onset reduced proteinuria, kidney damages, antibodies levels in the serum and ameliorated overall mouse survival. Despite the decreased in antibody levels in serum from Apophe-treated mice, we did not find any differences in the frequencies of plasmablasts/PC in spleen and BM of these mice compared to isotype-treated mice. This is consistent with the lack of decrease for plasmablasts/PC in *APRIL^−/−^* compared to control mice. The increased survival observed here is very similar to the APRIL/BAFF antagonism mediated with soluble TACI during treatment and at early time points after treatment stop (6 weeks) [10], [11], [12]. In this time frame, it is also very similar to the BAFF-R treatment [10], [11], [13], [14]. Later time points revealed a superiority of the TACI and BAFF-R over Apophe treatment. This might indicate that APRIL/BAFF blockade is superior to APRIL blockade. A conclusion that has some biological sense, even though pharmacokinetics of TACI-Ig and Apophe has not been compared. For BAFF-R, it is difficult to draw any conclusion since the delivery mode, BAFF-R-encoding retrovirus, was significantly different.

At the Ig level, APRIL blockade reduced IgA and IgM levels. For IgM, anti-dsDNA and anti-chromatin levels, and for IgG, anti-chromatin levels were also affected, constituting a molecular explanation to the disease amelioration observed. Although IgM autoantibodies have been considered as protective in lupus, total IgM and/or IgM anti-DNA are used as readout for disease activity in TACI-Ig or BAFF-R-Ig treated lupus mice experiments, and their reduction correlates systematically with disease amelioration [11], [14]. Furthermore, our ongoing experiments on CD22-deficient mice that develop lupus-like nephritis in association with the production of autoantibodies of IgM class (anti-DNA) rather suggest a pathogenic role of IgM autoantibodies in lupus.

By contrast, total and anti-dsDNA IgG were unaffected. Hence, mAb-mediated APRIL blockade largely reproduces the defect observed in APRIL-deficient mice by affecting mainly IgAs and IgMs [9]. Previous treatment of NZB/W F1 mice with an adenovirus secreting soluble TACI resulted in decreased IgM and IgG anti-dsDNA levels [11], [13]. In contrast, soluble BAFF-R decreased anti-DNA IgG, but had no effect on IgM in this model [13]. Taken together, this indicates that production of anti-dsDNA IgM and IgG may be more dependent on APRIL and BAFF, respectively. These highlight that auto-antibody production in NZB/W F1 is a complex process, implicating distinct molecular pathways. Notably, the anti-BAFF and anti-APRIL targeting trial taught us that it is not necessary to dampen different antibody subclasses to delay disease occurrence.

It is now clear that lupus pathology can be ameliorated by several B-cell targeting therapies, at least in the NZB/W mouse spontaneous model. While others have reported disease amelioration by affecting the mature B-cell repertoire without altering the autoantibody levels [10], we are showing here the opposite. Indeed, we obtained a similar delay in lupus occurrence, by reducing autoantibodies without affecting the host mature B-cell repertoire, as expected from the immune phenotype of APRIL-deficient mice. Hence, PC targeting by APRIL antagonism constitutes a novel attractive therapy for SLE patients. Future clinical trials should tell us whether mature B-cell or plasma-cell targeting is the best effective treatment with minimal adverse immunosuppressive effects.

## Materials and Methods

### Ethics statement

Animal experiments described in the present study have been approved by the Geneva ethics committee veterinarian office (protocol ID number: 1005/3536/3).

### Mice

Female NZB, male NZW and female NZB/W mice were purchased at the Jackson Laboratory. Female C57BL/6J (B6) mice were obtained from local breeding. B6.*APRIL* ^−/−^ mice were kindly provided by Dr Ashkenazi (Genentech). Proteinuria was tested in urine by Albustix reagent strips (Siemens Healthcare Diagnostics Inc.) every other week, and scored on a scale from 0 to 4 according to protein concentration (from 0 to ≥20 g/l).

### Anti-APRIL generation, production, and characterization

The IgG1 Apophe mAb was generated by immunizing B6.*APRIL^−/−^* mice subcutaneously with murine recombinant APRIL (Adipogen) in Stimune adjuvant (Prionics), and fusion of primed leukocytes from draining inguinal lymph nodes with the X63Ag8 myeloma cell line (American tissue culture collection, ATCC). The antagonism activity of Apophe for APRIL/BCMA or TACI interaction was tested in a standard ELISA assay. Briefly, huTACI-Ig or huBCMA-Ig (Adipogen, 1 µg/ml) were coated o/n at 4°C. Plates were blocked and the binding of flag-tagged muAPRIL fused to ACRP30 (APRIL/ACRP30, Adipogen, 300 ng/ml) or flag-tagged muBAFF (Adipogen, 300 ng/ml) was revealed with a biotinylated anti-flag (clone M2, Sigma) followed by steptavidin-conjugated horseradish peroxidasae (BD Biosciences). The antagonism activity of Apophe for APRIL/HSPG interaction was tested by flow cytometry with the myeloma L363 cell line (ATCC). Briefly, 1 µg/ml flag-tagged murine APRIL/ACRP30 or control ACRP30 (Adipogen) was incubated with L363 cells, and binding was revealed with biotinylated anti-flag, followed by steptavidin-conjugated phycoerythrin (BD Biosciences). Control Ig (cIg) was an IgG1 anti-huCD7 (clone 3a1, ATCC). Both mAbs were produced in serum-free medium and purified on protein-G sepharose. Absence of endotoxin contamination in purified mAb preparation was tested in a LAL assay (Lonza). For the MHC-class II upregulation test, total splenocytes were incubated with 1 µg/ml of murine APRIL (Adipogen) as previously reported [28]. After two days, MHC class II expression was monitored by flow cytometry on gated B220^+^ B cells with the mAb AF6-120.1 (BD pharmingen).

### Quantitative RT-PCR

RNAs from BM and spleen cells were prepared with TRIzol reagent (Invitrogen AG). APRIL mRNA was quantified by real-time RT-PCR with cDNA prepared from RNA. cDNA was amplified using the following primers: forward primer 5′-CTGGAGGCCAGGGAGACAT-3′ and reverse primer 5′-GCACGGTCAGGATCAGAAGG-3′. PCR was performed using the iCycler iQ Real-Time PCR Detection System (Bio-Rad) and iQ SYBR green Supermix (Bio-Rad). Results were quantified relative to a standard curve generated with serial dilutions of a reference cDNA preparation from spleen and normalized using TATA-binding protein (TBP) mRNA.

### Western blot analysis

Supernatant of BM flushes, obtained after centrifugation of whole tibiae and femurs in a fixed volume (100 µl) of PBS, were loaded onto 15% SDS-PAGE gel (20 µg protein per lane), and transferred to nitrocellulose membrane (Bio-Rad) with a semi-dry blotting apparatus (Bio-Rad). After blocking o/n at RT in 20 mM Tris-HCl (pH 7.4), 150 mM NaCl, and 0.1% Tween 20 containing 1% non-fat dry milk powder, membranes were incubated with the ED2 rabbit polyclonal anti-APRIL antibody (4 µg/ml) (ProSci) 1h30 at RT. Thoroughly washed membranes were incubated with HRP-conjugated goat anti-rabbit IgG (Bio-Rad) for 1 h at RT. Chemiluminescence development was conducted with the Amersham-ECL reagents (GE Healthcare Biosciences), and the membranes were exposed to HyperFilm ECL (GE Healthcare Biosciences).

### Histology

Kidney samples were collected when mice were sacrificed either at 8 months-old or at the end of the experiment. For immunofluorescence staining, kidneys were embedded in Tissue-Tek O.C.T. compound (Miles) and snap-frozen in liquid nitrogen. Four µm frozen sections were stained with Alexa Fluor 488-labelled anti-mouse IgG (Invitrogen), TXRD-labeled anti-mouse IgM (Southern Biotechnology) and FITC-labeled anti-mouse C3 (Cappel) antibodies in the presence of 2.4G2 anti-FcγRII/III mAb. For histopathology, sections were stained with periodic acid-Schiff (PAS) reagent. The extent of glomerulonephritis was based on the intensity and extent of histopathological changes, as described previously [29].

### Flow cytometric analysis

Flow cytometry was performed using three- or four-color staining and analyzed with a FACSCalibur (BD Biosciences). The following antibodies were used: anti-B220 (RA3-6B2), anti-CD4 (GK1.5), anti-CD8 (53-6.7), anti-CD138 (281-2), anti-CD43 (S7) (e-biosciences), anti- IgM (lomm9), and anti-IgDa (AMS15.1). Staining was performed in the presence of saturating concentration of 2.4G2 anti-Fc-RII/III mAb.

### Serological analysis

Serum levels of IgG autoantibodies against chromatin and dsDNA were determined by ELISA. Chromatin, prepared from chick erythrocytes was directly coated to ELISA plates, whereas calf-thymus dsDNA was coated to ELISA plates precoated with poly-L-lysine (Sigma-Aldrich). Plates were then incubated with 1/100 diluted serum samples, and development performed with alkaline phosphatase-labeled goat anti-mouse IgM or IgG. For anti-dsDNA and anti-chromatin antibodies, results are expressed in U/ml in reference to a standard curve obtained with a serum pool of MRL-*Fas^lpr^* mice. Serum levels of total IgA, IgG and IgM were quantified by ELISA and results are expressed in µg/ml or mg/ml by referring to a standard curve obtained with a mouse serum with known IgA, IgG and IgM concentrations (Miles Laboratories).

### Statistical analysis

The Kaplan-Meyer log-rank test was used for the statistical analysis of mortality rates. Analysis of serological parameters was performed by the Mann-Whitney *U*-test. Unpaired comparisons for mRNA expression and proteinuria levels were carried out by the Student's *t* test. Probability values <5% were considered significant.
